# 

**Published:** 1906-11-17

**Authors:** 


### BOOKS RECEIVED.

Baillière, Tindall, and Cox.

"The Sigmoidoscope." By P. Lockhart Mummery, F.R.C.S.

"Syphilology and Venereal Disease." By C. F. Marshall, M.D.

"Minor Maladies and their Treatment." By Leonard Williams, M.D.

"The Uses of Röntgen Rays in General Practice." By R. Higham Cooper, L.S.A.

"Manual of Bandaging." 6th edition. By C. H. Leonard, M.D.

Hodder and Stoughton.

"Physical Diagnosis." By E. Le Fevre, M.D.

"Dental Materia Medica." By E. H. Long, M.D.

Methuen and Co.

"The Hygiene of Mind." By T. S. Clouston, M.D.

J. Nisbet and Co.

"Experiments on Animals." By Stephen Paget.

S. Partridge and Co.

"The Zenana." Vol. 13, 1906.

Whittaker and Co.

"Dissections." Illustrated. By C. Gordon Brodie, F.R.C.S.

J. Wright and Co., Bristol.

"A Guide to Urine Testing." By M. Robinson, L.R.C.P.

"Women's Health: How to take care of it." 2nd edition. By Florence Stacpoole.

"Lectures on Massage and Electricity." 6th edition. By T. Stretch Dowse, M.D.

J. Bale, Sons and Danielson, Limited.

"A Few Hints on the Care of Children at Sea." By S. Synge, M.D.

"Pulmonary Phthisis." By H. Hyslop Thomson, M.D.

Cassell and Co., Limited.

"The Health Reader." By W. Hoskyns-Abrahall.

Funk and Wagnells Co.

"The Health Care of the Baby." By L. Fischer, M.D.

The Garden City Association.

"Housing in Town and Country." By T. Adams.

"The Garden City Movement." By G. Montagu Harris, M.A.

C. Griffin and Co., Limited.

"Outlines of the Diseases of Women." 4th edition. By J. Phillips, M.D.

J. Jacobs.

"Hypnotism and Suggestion." By Edwin Ash, M.B.

John Murray.

"Cancer of the Breast." By W. Sampson Handley.

His Majesty the King has sent his annual subscription of £100 to the funds of the Middlesex Hospital.

### NOTICE TO CORRESPONDENTS.

All MSS., Letters, Books for Review, and other matters intended for the Editor should be addressed The Editor, "The Hospital," The Hospital Buildings, 28 & 29 Southampton Street, Strand, W.C.

The Editor cannot undertake to return rejected MS., even when accompanied by stamped directed envelope.

**Notice to Advertisers and Subscribers.**

All Advertisements, Orders for Copies of The Hospital, and Business Communications should be addressed to **THE MANAGER (Not to the Editor),** The Hospital, 28 & 29 Southampton Street, Strand London, W.C.

The Cost of Subscription, post free, is as follows:—Per week, 3½d.; per quarter, 3s. 3d.; per half-year, 6s. 6d.; per annum, 13s. Foreign and Colonial:—Per annum, 19s. 6d., payable, in all cases, in advance.

**NOTICE.—Vol. XL. of "The Hospital" (April to September 1906), in handsome cloth binding will shortly be on sale at the office of this Journal price 10s. 6d. Cases for binding the Numbers contained in this Volume 2s. Index to Vol. XL., 3½d., post free.**

**The Monthly Part for November is now ready. Price 1s., by post, 1s. 3d.**

### CHARTS

### MORNING & EVENING

Arranged for four weeks.

### FOUR-HOUR CHARTS

For recording Temperatures at the hours of

**2, 6, 10, or 12, 4, 8,**

for a period of nine complete days (i.e., day and night).

Price ***6d. a dozen,*** or in quantities of

| | | |
|---|---|---|
| 25 ... ... ... | **1/0** | *Carriage Paid.* |
| 50 ... ... ... | **1/6** | |
| 100 ... ... ... | **2/9** | |
| 250 ... ... ... | **6/6** | |
| 500 ... ... ... | **12/0** | |
| 1,000 ... ... ... | **23/0** | |

These Charts are especially prepared for Institutions and Private Nurses to a standard size of 10 in. by 8 in. They are clearly printed on good paper, and will be found to be the cheapest and most serviceable Charts published.

**JAPANNED TIN WASHABLE CHART-HOLDERS.**

Specially designed for the above Charts. Stoutly made, easily cleaned or sterilized.

**1/-** each, **9/6** per dozen, carriage paid.

**A Useful Novelty for District, Private, and Maternity Nurses.**

**THE POCKET-BOOK OF TEMPERATURE CHARTS.**

Containing 17 Twelve-hour and 8 Four-hour Charts, perforated so that each Chart may be removed after use. Size 8 by 5. Can be easily folded and carried in the cloak or apron pocket.

**6d.** net, **7d.** post free.

### CASE-BOOKS

**A CASE-BOOK FOR PRIVATE NURSING.**

Arranged for Day and Night Nurses' Report, with rulings in accordance with what has been found in practice to be the most suitable and approved system for Pay Hospitals, Nursing Homes, Private Nurses, &c. The size of an Exercise Book, 7½ in. by 9½ in., containing 48 pp.

**This Case-book has met with the highest praise from foremost Members of the Medical Profession.**

**3d.** net, **4½d.** post free. Special quotations for a quantity.

**THE POCKET CASE-BOOK FOR DISTRICT AND PRIVATE NURSES.**

By a PHYSICIAN. Suitable size for apron pocket.

Cloth boards, **1/-** net ; in handsome leather, gilt, **1/6** net, **1/7** post free.

**THE "SIMPLEX" DISTRICT NURSES' CASE-BOOK.**

Containing space for 50 Cases, ruled and printed. Strongly bound in cloth boards. Size, 6 by 4.

**6d.** net, **7d.** post free.

***Complete Catalogue of Nursing Manuals, &c., post free on application.***

**The Scientific Press, Ltd.,**

**Publishers of Nursing Text-Books, Charts, and Case-Books of all Descriptions.**

**28 & 29 SOUTHAMPTON STREET, STRAND, LONDON, W.C.**

